# Search traffic for inflatable penile prosthesis increased following the COVID-19 pandemic in the United States: a Google Trends analysis

**DOI:** 10.1038/s41443-024-00922-6

**Published:** 2024-05-30

**Authors:** Elia Abou Chawareb, Hana Nakamura, Muhammed A. M. Hammad, Jake A. Miller, Supanut Lumbiganon, Babak K. Azad, Faysal A. Yafi

**Affiliations:** 1 Department of Urology, University of California, Irvine, CA USA; 2 Department of Surgery, Faculty of Medicine, Khon Kaen University, Khon Kaen, Thailand

**Keywords:** Surgery, Sexual dysfunction

## Abstract

We aimed to determine the impact of the COVID-19 pandemic on search trends for inflatable penile implants in the US. Search trends for inflatable penile implants ranging from 2016 through 2023 were analyzed utilizing Google Trends. Associations between search trends and US Census Bureau data, including average household income and per capita income, were analyzed. Pre- and post- COVID-19, the interest in inflatable penile implants has been steadily increasing on average in the US. The average household income for counties with the highest interest in inflatable penile implants during the pre-COVID era was $53,136, whereas for the counties with the highest interest in inflatable penile implants in the post-COVID era, the average decreased to $50,940. Similarly, the average per capita median decreased from $35,209 to $34,547. Search traffic for inflatable penile prosthesis increased following the pandemic in the US. Nevertheless, post-pandemic, individuals with lower income levels showed no change in interest in penile implant searches compared to the pre-pandemic period. Understanding this steadiness in interest can inform healthcare professionals and policymakers to tailor interventions and educational efforts to reach a broader audience, ensuring equitable access to information and healthcare resources.

## Introduction

Erectile dysfunction (ED) is estimated to affect around 40% of U.S. men, with upwards of $190 million reimbursed annually by Medicare for its medical management alone [1]. Management for ED most commonly includes the use of oral medications, and intracorporeal injections [2]. However, for patients with ED refractory to medical management, the surgical insertion of a penile prosthesis remains the gold standard to many urologists [3]. Yet, the fields of urology and andrology persist in advancing ED treatment to become more inclusive across a broader range of patients. Patients who may not be suitable candidates to the aforementioned treatments, or who do not find them effective, may explore newer treatment options such as shockwave therapy, stem cell therapy, and platelet-rich plasma (PRP) therapy [4]. Among these, the inflatable penile prosthesis (IPP), the most common type of penile implant, stands out as the most popular treatment choice offered to individuals with a more severe condition [5].

The COVID-19 pandemic presented the US healthcare system with challenges, including increased expenses for drugs, supplies, and staff, alongside decreased revenues due to canceled visits and surgeries [6, 7].

Healthcare providers sought to maintain continuity of care through telemedicine and online services, which were well-received by patients. As a result, these services continue to be utilized post-pandemic [8, 9]. Recognizing the substantial influence of the pandemic on society and healthcare, this study aims to explore the impact of the COVID-19 pandemic on public interest in and search trends for IPP in the US.

## Materials and methods

### Google Trends

Google Trends, a website created by Google Inc., provides open and free access to trending search queries worldwide by providing insights into the popularity of these queries on Google’s search engine. It also offers a time analysis feature, allowing users to examine search data spanning from years ago to the present day [10]. This tool continues to serve as a robust resource in interdisciplinary studies, enabling the analysis of historical trends, pattern identification, and even predictions of future outcomes, such as viral outbreaks [11]. In the healthcare context, Google Trends has become a valuable tool for monitoring and analyzing public interest and search patterns related to health topics. It allows healthcare professionals, researchers, and policymakers to gain insights into evolving health trends, public concerns, and the impact of health-related events or interventions [12].

**Fig. 1:**
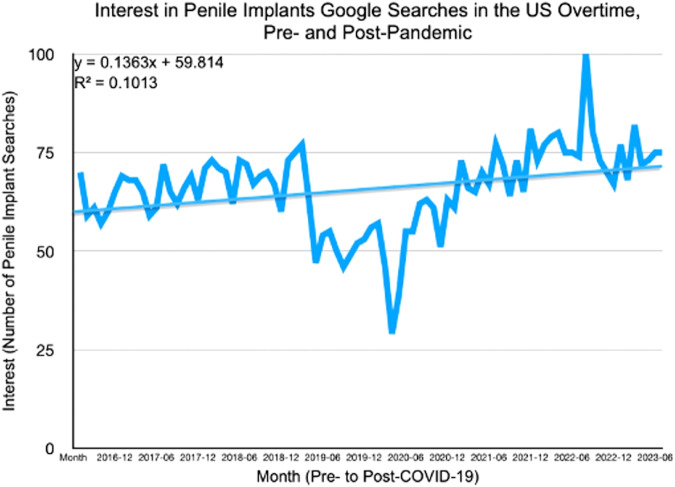
Interest in inflatable penile implants Google searches in the US over time, pre- and post-pandemic.

Google Trends was used to analyze data encompassing pre-pandemic, post-pandemic, and during the pandemic periods. The “pre-pandemic” phase, defined as the two-year period from March 15, 2018, to March 15, 2020, was selected to encapsulate data up until the point when the US initiated widespread school district closures and entered an officially mandated quarantine period [13]. The post-pandemic period was defined to be from June 15, 2021, to June 13, 2023. This timeframe was selected to maintain an equal two-year period before and after the pandemic for a comparable dataset. The Google Trends search interest numbers are expressed relative to the highest point of popularity for the most frequently searched term within the specified query, considering both the region and time. Ranging from 0 to 100, a value of 100 denotes peak popularity, while a value of 50 signifies that the term is half as popular at that particular time point. Subsequently, these values were normalized in relation to control search terms specific to the given region and time, and they are presented as arbitrary units (a.u.) spanning from 0 to 100 a.u. [14]. To address for originally large fluctuations in data points, multiple search terms for IPP were considered to accommodate for such varying data. This approach allowed for more comprehensive datasets, considering natural human differences. The search terms “inflatable penile prosthesis,” “IPP,” “IPP implant,” and “penile implant” were analyzed, then the raw data was combined and averaged to determine the impact unit per week across all four terms, resulting in the graph presented as Fig. 1.

### US Census Bureau

Data from the US Census Bureau [15] was analyzed to determine the average household income and average per-capita income for the years 2018, 2019, 2021, and 2022. The median incomes for the year 2020 were excluded from the analysis, as it represents the onset of the COVID-19 pandemic and serves as the dividing point between the pre- and post-COVID eras. Google Trends data automatically identified metropolitan “hot spots” in which searches for IPP were the most abundant. We selected the five metropolitan areas or counties in the US with the highest interest in IPP in the pre-COVID era and similarly in the post-COVID era. Income data for these top metropolitan “hot spots” was collected based on the US Census Bureau data.

## Results

From 2016 to 2023, the overall search interest in IPP exhibited a consistent and upward trend in the US (Fig. 1). From 2018 until the onset of the COVID-19 pandemic, there was a consistent upward trajectory, suggesting a growing interest in IPP. However, a notable decline is evident during the pandemic period, indicating a temporary dip in interest. Post-pandemic, starting from 2021, there is a significant resurgence in interest, marked by an upward trend. Despite fluctuations, the overall slope demonstrates an increasing pattern. Within these interest groups, a select number of metropolitan areas and counties emerge as the most significant contributors for the most IPP searches and interest within the USA.

Before the onset of COVID-19, the search hotspots for IPP were notably concentrated in metropolitan areas characterized by a relatively higher 2021 median household income and per capita income. These areas included Birmingham, Alabama; West Palm Beach-Ft. Pierce, Florida; New Orleans, Louisiana; Orlando-Daytona Beach-Melbourne, Florida; and Nashville, Tennessee. After COVID-19, these search hotspots remained relatively similar, with some notable shifts to areas like Mobile, Alabama; and Tampa-St. Petersburg (Sarasota), Florida, replacing Orlando-Daytona Beach-Melbourne, Florida; and Nashville, Tennessee.

**Fig. 2:**
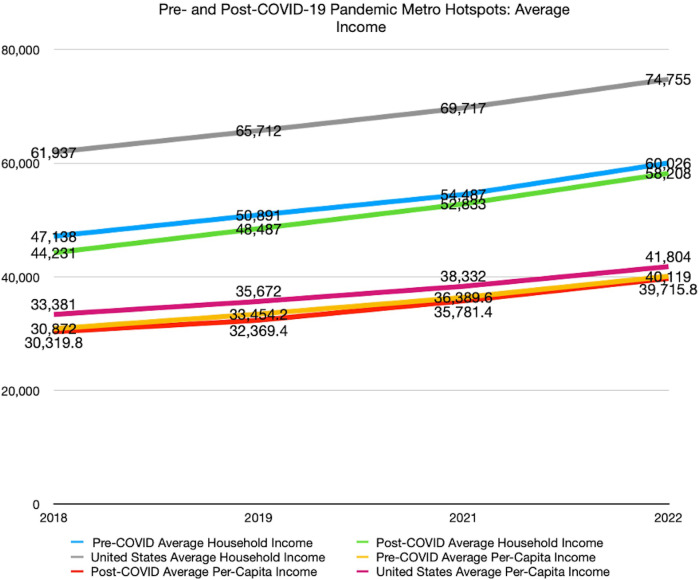
Linear model showing pre- and post-pandemic metro hotspots’ average household income and per-capita income.

**Fig. 3:**
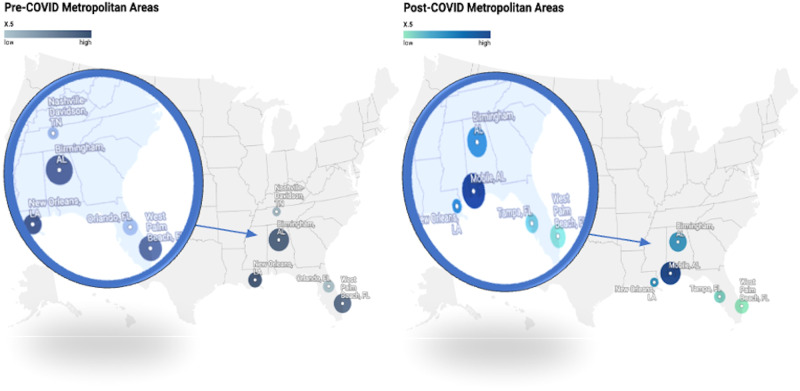
The top 5 metropolitan areas displaying the most significant search interest before and after COVID-19 pandemic.

Counties with highest search interest before the COVID-19 pandemic had an average household income of $53,136 in the years 2018 till 2022. Simultaneously, the per capita median income was $35,209. On the other hand, the counties with highest search interest after the COVID-19 pandemic exhibited lower averages. The average household income decreased to $50,940, while the per capita median income decreased to $34,547 (Figs. 2 and 3). However, the changes in both averages in the US over the years were not statistically significant, as indicated by p-values of 0.61 for household incomes and 0.82 for per capita income.

## Discussion

To our knowledge, this is the first study to use internet search intent to measure the interest in IPP in the United States. Despite the wealth of studies delving into the trends of IPP implantation, a distinct gap exists in the quantification of public interest in this particular procedure [16, 17]. Our findings revealed a steady increase in IPP interest among the nation’s population over the years. Numerous theories can explain the overall surge in Google search trends for IPP procedures after the COVID-19 pandemic in the US. One plausible explanation could be linked to the increased awareness and the continuous evolution of technological and clinical practices of the procedure. Moreover, these advancements allowed the possibility for designing unique IPPs for special populations, including transgender men [18]. This technical and clinical progress might, in turn, lead to an increased public exploration of the subject matter [17]. Additionally, lifestyle changes and external factors such as libido, psychological stress, and the questionable relationship between COVID-19 and ED during and/or after the pandemic likely contributes to the intensified search interest [19, 20]. The compounded impact of COVID-19 and psychogenic disorders stemming from isolation, grief, and anxiety could contribute to the increased prevalence of ED, which may be further exacerbated by the emergence of pre-existing sleep disorders or those induced by the pandemic [20]. Another hypothesis on the relationship between COVID-19 and ED is shown by the lower testosterone and higher LH levels found in reproductive-aged SARS-CoV-2-infected males compared to healthy controls [21]. Rastrelli et al. associated SARS-CoV-2 infection with decreased total and free testosterone levels, positively correlating with ED [22]. Additionally, the pandemic’s impact on mental health, particularly anxiety and depressive symptoms, has been consistently linked to a decline in male sexual function, including erectile response [23]. Lower psychological adjustment during imposed confinements has been shown to negatively affect erectile function, sexual desire, and overall satisfaction in men [24]. Moreover, we can hypothesize that the surge in attempts of sexual intercourse during confinement may have brought attention to underlying ED, particularly in men experiencing decreased sexual desire [25]. Notably, searches for ED witnessed a discernible spike during the pandemic in the US, suggesting a potential impact of the psychological burden imposed by the global crisis [26]. The number of searchers may have increased proportionally with the number of people experiencing ED symptoms, leading to the desire to study the different treatment options. This also aligns with a broader societal shift wherein many Americans, especially those without insurance or immediate healthcare access, prefer utilizing the Internet as a primary source for addressing medical concerns [27].

However, the observed decline in interest during the pandemic can be ascribed to a shifting healthcare landscape, where priorities skewed towards COVID-19 patients and economic uncertainties likely impacted the accessibility of IPPs. Also, internet searches were mostly concentrated on the COVID-19 pandemic updates, prevention, and management [28]. Furthermore, healthcare resources and research were primarily directed towards addressing the pandemic. Simultaneously, elective surgeries were delayed and deferred [29]. Nevertheless, the post-pandemic period witnessed a significant resurgence in interest, with an increased interest among lower-income groups compared to the pre-pandemic era in the US. This resurgence in interest can be linked to a variety of factors, including increased overall awareness of the procedure, a growing number of physicians specializing in IPPs, targeted advertisements, and enhanced accessibility. The latter is further facilitated by the growing prevalence of telemedicine, offering a more accessible avenue for healthcare consultations [9].

Counties exhibiting the highest interest in IPP during the pre-pandemic era demonstrated similar average household and per-capita incomes over the years compared to those with the highest interest during the post-pandemic era in the US. This stability indicates a non-growing interest in IPP within communities with lower incomes, potentially associated with a steady emphasis on investment in men’s health. The high interest in these affluent areas may also be a reflection of the relatively higher cost of IPP implantation treatments. Furthermore, there was a notable rise in healthcare access disparity, particularly for elective surgeries, in the post-pandemic period [30]. Furthermore, we observed that metropolitan areas with the highest search interest are primarily concentrated in the Southeast region rather than in densely populated areas, suggesting that less populated areas may have less knowledge about IPP. This trend could be attributed to factors such as increased advertising and easier access to care in larger, more populated cities, leading to higher search volumes for IPP in smaller, less populated cities. However, additional studies are needed to support the attribution.

The multifaceted nature of these factors highlights the intricate interplay between societal trends, healthcare accessibility, and technological advancements in influencing the trajectory of public interest in IPP procedures. Nevertheless, our results showed an increased surge in interest regarding the procedure in the post-COVID era compared to the pre-COVID era. This upward trend signifies a growing popularity of the operation, which could hypothetically be attributed to its success and the high satisfaction reported by both patients and their partners [31, 32].

Our study acknowledges certain limitations. The reliance on internet data introduces potential influences from various unknown factors, such as media exposure or alterations by users or bots. Google Trends lacks detailed demographic information about the users included in our study, preventing a comprehensive analysis of user characteristics. Also, the geographic analysis is limited by the heterogeneity within large regions, and smaller subdivisions were not feasible due to data limitations. Moreover, users may have utilized synonyms for IPP that were not captured in our data collection. Additionally, the search volume data provided by Google Trends represents relative search volume normalized to the total number of searches on Google, leaving the absolute number of users or searches unknown. Furthermore, the study’s time series analysis acknowledges the possibility of concurrent events influencing the observed changes in public interest.

## Conclusion

This study demonstrates an increase in Google search trends for IPP procedures following the COVID-19 pandemic in the US. Nevertheless, post-pandemic, individuals with lower income levels showed no change in interest in penile implant searches compared to the pre-pandemic period. This lack of shift could be attributed to reduced investment in men’s health within this demographic. Understanding this steadiness in interest can inform healthcare professionals and policymakers to tailor interventions and educational efforts to reach a broader audience, ensuring equitable access to information and healthcare resources; a crucial aspect in the domain of men’s health, where disparities in access are still prevalent.

## Data Availability

The datasets during and/or analyzed during the current study available from the corresponding author on reasonable request.
